# Organoids to Remodel SARS-CoV-2 Research: Updates, Limitations and Perspectives

**DOI:** 10.14336/AD.2023.0209

**Published:** 2023-10-01

**Authors:** Yucheng An, Yanjie He, Nan Ge, Jintao Guo, Fan Yang, Siyu Sun

**Affiliations:** ^1^Department of Gastroenterology, Shengjing hospital of China Medical University, Shenyang, China; ^2^Department of Surgery, New York University School of Medicine and NYU-Langone Medical Center, New York, NY, USA

**Keywords:** severe acute respiratory syndrome coronavirus 2, organoid, mechanism, drug and vaccine screening

## Abstract

The novel COVID-19 pneumonia caused by the SARS-CoV-2 virus poses a significant threat to human health. Scientists have made significant efforts to control this virus, consequently leading to the development of novel research methods. Traditional animal and 2D cell line models might not be suitable for large-scale applications in SARS-CoV-2 research owing to their limitations. As an emerging modelling method, organoids have been applied in the study of various diseases. Their advantages include their ability to closely mirror human physiology, ease of cultivation, low cost, and high reliability; thus, they are considered to be a suitable choice to further the research on SARS-CoV-2. During the course of various studies, SARS-CoV-2 was shown to infect a variety of organoid models, exhibiting changes similar to those observed in humans. This review summarises the various organoid models used in SARS-CoV-2 research, revealing the molecular mechanisms of viral infection and exploring the drug screening tests and vaccine research that have relied on organoid models, hence illustrating the role of organoids in remodelling SARS-CoV-2 research.

## 1. Introduction

In December 2019, cases of pneumonia of unknown aetiology were reported in Central China. On 7^th^ January 2020, the culpable pathogen, which had spread worldwide through human-to-human contact, as suggested by familial cluster studies [1, 2], was isolated and identified as the severe acute respiratory syndrome coronavirus 2 (SARS-CoV-2) virus. Since then, over 600 million people have been diagnosed with coronavirus disease 2019 (COVID-19), and over 6 million patients have died from it, making it a grave threat to human health. Manifestations of the disease listed in order of frequency include fever, cough, dyspnoea, headache, and diarrhoea. Shock, acute respiratory distress syndrome (ARDS), arrhythmia, and acute cardiac trauma are the main complications observed, while in a cohort study of 1733 discharged patients, fatigue, myasthenia, and dyskinesia were shown to be the main sequelae [3-5]. However, the large number of COVID-19 infections, complex symptoms, as well as the emergence of Omicron variants and other mutants have hindered COVID-19 research. To date, despite the efforts of the global scientific community to trace the origin of the virus and develop effective drugs and vaccines, the virus has been mutating faster than the speed of ongoing research.

SARS-CoV-2 belongs to the genus *Betacoronavirus* and is phylogenetically close to SARS-CoV-1 in terms of the spike protein [6, 7]. The S protein is essential for the host invasion of the SARS-CoV-2 virus, with the activation of the membrane fusion relying on the cleavage of the S protein. TMPRSS2 and cathepsin L are of vital importance in this process. The S protein is cleaved into 2 subunits, S1 and S2. Following a complicated conformational transition, the S1 and S2 subunits transform into their active forms, mediating membrane fusion. The S1 subunit carries the receptor binding domain (RBD) that recognizes and binds to the angiotensin-converting enzyme 2 (ACE2), which has been shown to be the receptor for SARS-CoV-2 [8-10]. The Omicron and Delta variants have spread to more than 38 countries, and such rapid mutation rates and strong infectivity have prompted scientists to search for mutants. Mutations of the S protein RBD on the Omicron and Delta variants have a greater affinity for ACE2 and might have increased the infectivity of the virus several times [11, 12]. Briefly, binding of the S protein to ACE2 induces membrane fusion, with TMPRSS2 and cathepsin L further promoting this process. Accordingly, mutations of the S protein and RBD have led to the emergence of mutants with stronger infectivity.

ACE2 is an important balancing factor in both the renin-angiotensin-aldosterone system (RASS) and Kinin-Kallikrein system (KKS). Of note, RASS provides a suitable environment for vasculature by preventing inflammation and fibrosis and converts AngII into Ang1-7 to prevent vascular damage.

While, KKS plays an important role in inflammation, thrombogenesis, and vascular physiology, mainly realizing the strong vascular dilation function against RASS [13]. ACE2 is commonly expressed on various organs in the human body, such as the lungs, heart, brain, intestinal tract, kidneys, and blood vessels [14]. A study showed that ACE2 (and its variants) is the key receptor of not only SARS-CoV-2, but also a crucial factor in the development of other diseases, such as hypertension [15]. The level of viral load has been closely related to the level of expression of ACE2 on targeted cells. In addition, released inflammatory factors upregulate the levels of ACE2, forming a positive feedback between the levels of ACE2 and those of inflammatory factors [8]. Downregulation of the levels of ACE2 after SARS-CoV-2 infection leads to AngII accumulation, finally causing inflammation, fibrosis, and vasoconstriction. Similarly, an imbalance in KKS caused by ACE2 internalization was reported to enhance the vascular inflammatory response, leading to pulmonary oedema [13]. Considering its important role in SARS-CoV-2 infection, targeting ACE2 would be an appropriate therapeutic approach. We might inhibit the expression of ACE2 on the cell surface, or neutralize the virus by supplying an alternative receptor, such as human soluble ACE2, chondroitin sulfate, or neuropilin 1 [16].

An increasing number of studies, using various models, has been conducted to understand and overcome COVID-19. Cell lines, such as primary human airway epithelial cells, infinitely proliferative cells, such as Calu-3 cells, and the most commonly used Vero E6 cells, have all been used in various studies. Animal models used include Syrian hamsters, mice, ferrets, and rhesus macaques [17, 18]. However, limitations still exist in the use of these models: a single-cell line cannot reveal the systematic situation and cannot reflect the interactions between different cell types. Most cell lines are derived from malignant cells, and hence their indeterminate growth characteristics differ significantly from those of normal human cells, hindering the effective simulation of the response to drug screening; thus, tests conducted on 2D cell lines still need to be repeated and their results verified on animals or humans. However, both the outcomes of drug screening and mechanistic research using live animal models require a longer time to conclude. Moreover, the high cost associated with the care of live animals also limits their usage. The increased time required and related high economic costs made us consider whether there are other better experimental models. Moreover, the 3R principle of medical ethics requires that researchers reduce the use of animals and replace them with other non-animal models [19-21]. Therefore, creating a model that is more closely related to human physiology was urgently needed for future research.

## 2. Brief Introduction to Organoids

Organoids, a new type of 3D cell model, can perfectly solve the dilemmas presented by previous research methods and fuel new research on SARS-CoV-2. Organoids can be described as mini-organs that closely resemble the complexity, organization, and functions of cells in the human body compared with traditional 2D cell models. The culture cycle is approximately a few months, shorter than that required by live animal models, the generated quantity satisfies the abundant demand for the conduct of various experiments, and there are no ethical limitations for the use of organoids. The differentiation of stem cell-derived organoids can be regulated by adding specific combinations and concentrations of growth factors and signalling molecules, which determine the success or failure of the generated organoid [17, 18, 22]. According to the cell source, organoids can be divided into pluripotent stem cell-derived organoids and tissue stem cell-derived organoids; pluripotent cells include both induced-pluripotent stem cells and embryonic stem cells. Pluripotent stem cell-derived organoids exhibit a triploblastic structure, reflecting cell complexity in vivo, whereas tissue stem cell-derived organoids show a more mature cell environment without a mesenchymal structure. Both have their advantages; pluripotent stem cell-derived organoids permit accurate gene knockout and editing to explore individualized viral variations, and can be easily cultured in a large scale, hence permitting large scale screening or other experiments. Whereas, the cell source of tissue stem cell-derived organoids, which exhibit better maturation, is more easier obtained [23]. To date, organoids have been applied in the study of infections, genetic diseases, regenerative medicine, developmental biology, and anti-tumour therapy. Since the first organoid model reported in 2009, researchers have derived organoid models of various organs using stem cells from humans and animals, greatly facilitated the progress in infection studies [24]. Krenn et al. revealed the relationship between microcephaly, the Zika virus, and HSV-1 infection using human brain organoids [25]. A brain organoid was also used for the study of prion diseases, where it showed immune responses and individualized pathology changes with prion challenging. In addition, changes in cellular metabolism and cytokine secretion were also observed [26]. Holthaus et al. found that the cAMP/PKA signalling pathway was the main pathway involved in the damage of the intestinal epithelium barrier, as a result of infection by *Giardia duodenalis* [27]. Enteroids and induced intestinal organoids have also been demonstrated to be efficient model systems for the study of salmonella infections [28]. Research of infectious diseases involving organoids encompasses the study of viruses, bacteria, and parasites, aiming in the development of the medical field. Brain organoids have been utilised in the study of several neurodevelopmental disorders; for instance, the TREX1 gene was reported to be related to microcephaly [29]. In the field of anti-tumour therapy, organoids have been used for the generation of expanded iPSC CAR-T cells, which are more functional than traditional CAR-T cells [30]. Studies using mouse and human pancreatic ductal organoids determined the upregulated genes in pancreatic ductal adenocarcinoma (PDA) and successfully simulated its progression, indicating organoids as a vital model for PDA research and the development of new drug targets [31]. Moreover, gene editing using CRISPR/Cas9 technology has been applied to intestinal organoids for the study of viral replication and the delineation of the function of specific genes. Benign to malignant transformation has also been achieved using CRISPR/Cas9 technology in intestinal organoids [32, 33]. In summary, organoids are a widely used model that has been shown to be quite efficient in mechanistic, treatment, and drug screening studies. We thus believe that organoids could be feasible models for future medical studies.

**Figure 1.**
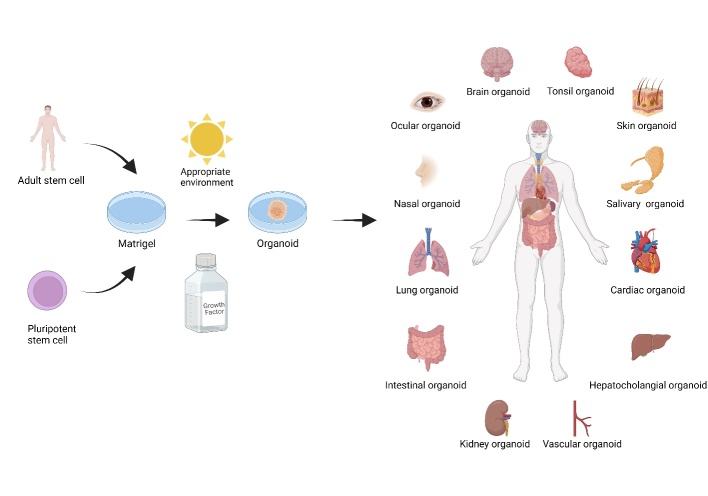
Adult stem and pluripotent stem cells are the main cell sources in organoid cultures. Both Matrigel and growth factors are also essential in organoid cultures. Organoid models of various organs have been used for studying the SARS-CoV-2 infection process.

This review aims to discuss the various applications of organoid models in SARS-CoV-2 research, including the mechanism of viral invasion into various organs, damage to host cells, host cell response, high-throughput drug screening based on various organ models, and some newly discovered potentially effective therapeutic targets (Fig. 1). We hope to clarify the entire pathogenic process of SARS-CoV-2 infection and determine a reasonable therapeutic approach. Concomitantly, we also summarize the modelling methods used for the generation of the corresponding organoids of each organ and compare them in an attempt to identify the more reasonable modelling approach.

## 3. Organoids tracing SARS-CoV-2 Origins, Mutations, and Species Specificity

At the time of the publication of this study, the origin of SARS-CoV-2 remains unclear. Different studies have reached different conclusions regarding environmental metabolite changes, viral genome traceability, and viral variations. Organoid models have shed some light on this matter. Zhou et al. tested bat intestinal organoids derived from crypts of Rhinolophus sinicus (Chinese horseshoe bats). This organoid contained basic intestinal cells, such as goblet cells and Paneth cells, and was infected by isolated SARS-CoV-2. The obvious increase in the viral load indicated that the bat intestinal organoids were permissive to SARS-CoV-2 infection. Immunostaining showed that the distribution of ACE2 and TMPRSS2 in the bat intestinal organoids was similar to that in humans. In addition, the bat ACE2 mRNA showed a high degree of homology to that of humans. This study established a bat gut organoid culture for the first time, and studies in both bat and human gut organoids provided the first experimental evidence that SARS-CoV-2 might have originated in bats [34]. However, defects in the above organoid hindered its long-term stability. Elbadawy et al. solved this problem by establishing a rousette bat intestinal organoid, in which Wnt3a, Noggin, R-spondin, EGF, FGF2,7,10, IGF, and TGF-α were added to the medium to maintain the active proliferation of cells [35]. An organoid study on the SARS-CoV-2 mutants Omicron and Delta indicated that Omicron showed lower infectivity in intestinal organoids, causing less damage, and resulting in a less obvious inflammatory response. In contrast, the Delta mutant acted similarly to the primary strain. We hypothesised that the decreasing tropism of intestinal cells was related to the mutation of the S protein of Omicron [36]. Another study indicated that Omicron entered host cells in a different manner because of the contradiction between the low affinity for ACE2 and the high susceptibility of host cells [37]. Interferon-induced transmembrane proteins (IFITMs) have been shown to be a vital cofactor for SARS-CoV -2 replication, including the main mutants on the 3D alveolospheres derived from human-induced alveolar type 2 cells. Species specificity was reflected in the dependence of IFITMs on several mutants. For example, the Alpha variant was reported to depend less on IFITM2 than other variants, whereas the Omicron variants are more dependent on IFITM1 and IFITM3 than other variants. It is therefore important to consider these differences among variants if we regard IFITMs as a new target for treatment, as different mutants might require different IFITMs as their main targets [38].

## 4. Organoids: a new window into SARS-CoV-2 research and a remodelling of the understanding of its pathogenic mechanism among different systems

Because of the biological characteristics of the 3D culture system and their stem cell origin, cells in organoid models have the characteristics of spontaneous organisation, proliferation, and differentiation, resulting in the formation of a tissue structure whose morphology mimics in vivo conditions. Therefore, compared with animal models, human-derived organoid models can often yield results that are closer to the actual in vivo state. In recent years, organoids have been used as disease models for studying a variety of infectious diseases, including influenza, norovirus infection, inflammatory bowel disease, and hepatitis B. Using organoids, it is also possible to simulate and study the specific process involved in a SARS-CoV-2 attack on the human body. Organoid studies of different human systems have shown the different levels of damage caused by SARS-CoV-2 and their specific mechanisms.

### 4.1. Respiratory System Organoids

Respiratory symptoms, including fever, cough, and dyspnoea, are common in patients with COVID-19; for example, bilateral ground-glass opacities can be observed on chest CT scans. In post-mortem examination of patients infected with SARS-CoV-2, diffuse alveolar damage was a common pathological change in the alveoli of all patients, including changes in the exudative and proliferative phases, which mainly cause capillary congestion, necrosis, and oedema of pneumocytes, hyaline membrane formation, and thrombogenesis [39]. An increasing number of studies has indicated that the level of inflammatory mediators was related to the mortality and severity of COVID-19 infection. The cytokine storm is thought to be responsible for the above pathological changes and the serious consequences and poor prognosis of patients with COVID-19 [40].

The nasal epithelium seems to be the first site of the respiratory system in the process of SARS-CoV-2 infection, and key permissive factors of infection, including ACE2, TMPRSS2, and furin, were shown to be highly expressed in the whole respiratory tract. SARS-CoV-2 RNA was detected in ciliate cells during the primary stage of COVID-19, with viral replication actively and simultaneously occurring in multi-ciliated cells [41, 42]. Research on this important site involved in the infection process, is therefore necessary. Zhou et al. used stem cells isolated from the turbinal tissue to establish a human nasal organoid, which contained a cell-stratified structure similar to that of human pseudostratified, ciliate, and goblet cells. The organoid was successfully challenged with the SARS-CoV-2 virus, and robust ciliate cell damage was observed, with the depth of infection even reaching the basal cell level [43]. During the infection process, the levels of pro-inflammatory factors, such as IL-6, IL-8, and VEGF-α were upregulated, which was thought to be the reason for the observed ciliate cell damage. Drug tests were also conducted using this human nasal organoid. In particular, 0, 80, and 640 μg/mL palivizumab were administered to 3 groups of respiratory syncytial virus (RSV)-infected models, respectively. The viral load in the 0 μg/mL group was almost 10^4^ times higher than that in the 640 μg/mL group, indicating that palivizumab was an effective drug against RSV and the potential use of the nasal organoid as an effective model for drug testing in future studies [44]. A recent study adequately explained the mechanism of viral invasion and ciliate cell damage using a nasal epithelium organoid model in air-liquid-interface cultures. In the initial phase of SARS-CoV-2 infection, the virus attached to cilia through ACE2 and TMPRSS2 on cilia surface. The following transportation of the virus from the cilia tip to the cell body and its mucociliary transportation facilitated viral dissemination in the nasal epithelium. In addition, Omicron variants showed a higher efficiency in both invasion and replication than D614G and Delta variants [45].

Compared with traditional 2D cell lines, 3D models better recapitulate the structure, functions, microenvironments, and morphologies of alveolar and other airway epithelium structures [46]. Thus, lung organoids have become an important tool in COVID-19 research. At present, there are 3 main classifications of lung organoids: bronchial, alveolar, and airway organoids.

### 4.1.1. Bronchial Organoids

**Figure 2.**
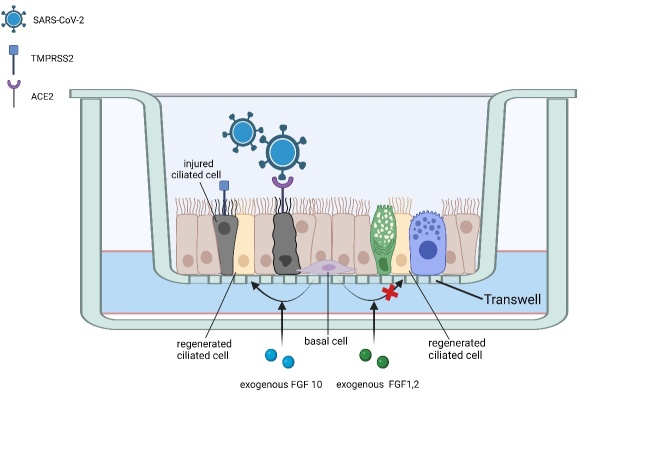
A BO-ALI model for investigating the main target cells during SARS-CoV-2 infection. This model indicated ciliated cells as the main cell type target of SARS-CoV-2 in bronchi. Following damage by inflammatory factors, basal cells regenerate the epithelium by differentiating into ciliated cells. FGF10 is the key factor in the regeneration process and could serve as a potential target for the treatment of respiratory epithelium damage. This organoid model also showed a higher rate of infectivity by SARS-CoV-2 than the BO model alone.

Bronchial organoids (BO) have been shown to be an effective model for SARS-CoV-2 studies. In the analysis of data from the Integrated Gene Expression System, 966 differentially expressed genes were identified from 3 infected and 3 uninfected BO samples. The expression of genes related to leukocyte migration, cell chemotaxis, and response to lipopolysaccharide was upregulated, which might be related with inflammation. Similarly, the expression of *CXCL1*, *EGF*, *CXCL10*, *CXCL8*, and *CCL5* was also upregulated; both CXCL8 and CXCL10 have been suggested to lead to lung damage, and CCL5 was shown to induce the migration of white cells to inflammation regions. These findings were similar to those observed in in vivo models, indicating that BOs can be used as an in vitro model for the study of SARS-COV-2 [47]. ACE2 and TMPRSS2 are highly expressed on the BO surface, with ciliate cells on the BO epithelium expressing high levels of ACE2, indicating them as the main targets of SARS-CoV-2 infection in human bronchi [48]. To improve traditional BO models, Sano et al. applied a microfluidic device in the BO to recapitulate the airflow in the human respiratory tract; this BO-ALI model was more likely to be infected by SRAS-CoV-2 than the BO alone. Tests using the BO-ALI model also showed that ciliated cells were the main target of infection, sustaining the most injury (Fig. 2). However, they also found that basal cells, which were not damaged by the virus, differentiated into ciliated cells to regenerate the epithelium. FGF10 is a key factor in this differentiation process and might therefore be related to post-infection regeneration of the bronchial epithelium [49]. As CXCL8, CXCL10, and EGF are hub genes in the infection process, studies on these genes might promote the development of SARS-CoV-2 research [47].

### 4.1.2. Alveolar Organoids

Human alveolar organoids (ALO) contain AT1 and AT2 cells; low levels of ciliated cells, goblet cells, and club cell markers were also detected. In addition, ACE2 and TMPRSS2 were found to be expressed on the cell surface, confirming their permissive nature for SARS-CoV-2 [48]. The inflammation response is known to be the primary cause of alveolar injury, and most pro-inflammatory and immune-related genes, including CCL5, IFN, TNFα, IL-6, and IL-8, were shown to be upregulated [50]. A study using an iPSC-derived alveolar organoid showed that AT2 cells were the centre of the inflammatory reaction, in which the NF-KB pathway was upregulated. The ligand and target of IFN were also upregulated after SARS-CoV-2 infection of the alveolosphere [51]. Interestingly, treatment with exogenous IFN after infection upregulated the expression of ACE2 in this model, whereas pre-treatment with IFN inhibited viral replication. However, during the viral infection process in this model, lower expression of interferon stimulating genes (ISGs) and levels of type IFNI or III ligands were observed in infected cells than uninfected cells, indicating that the IFN pathway was not as robust as we thought. This suggested the administration of exogenous IFNs as a novel therapeutic approach in the case of a compromised IFN response against viral infection [51, 52]. The differential potency of AT2 cells was demonstrated in an alveolar organoid derived from AT2 cells. Chiu et al. isolated immature AT2 cells from a lung organoid and performed the original alveolar organoid culture. After expansion for several months, AT2 cells developed into an alveolar sac, with AT1, AT2, and club cells being also detected. The composition ratio of each cell type was similar to that of stem cell-derived ALO, suggesting that AT2 cells have the ability to differentiate, and might serve as progenitor cells in ALO [53]. In another interesting study, ACE2 and TMPRSS2 were significantly upregulated by dPM2.5. The study was conducted using an hPSC-derived alveolar organoid, and suggested that long-term exposure to PM2.5 might increase susceptibility to SARS-CoV-2. A number of pathways related to fibrosis were also involved in this process, such as the ERK and TGF-β1-mediated EMT signalling pathways, which might explain lung fibrosis in patients with COVID-19 [54].

### 4.1.3. Airway Organoids

Airway organoids (AWO) contain the proximal respiratory tract and distal alveolar sac and can thus be described as a more complete model of the respiratory tract compared with ALO and BO. AWO has been proven to better reflect SARS-CoV-2 infectivity and host defence responses and is based on an easily obtainable cell source. Bronchoalveolar lavage fluid is an excellent source, resulting in a final organoid model that is similar to bronchial and alveolar epithelium, with pseudostratified, club, ciliated, basal, AT1, AT2, and goblet cells [55, 56].Accordingly, 2 studies carried out on AWO have made important contributions in the field of viral tropism. All 7 cell types were infected with SARS-CoV-2; ciliated cells were the main target in the proximal region, whereas AT2 cells were the main target in the distal alveolar region. Club cells, a newly detected potent cell target, were also shown to be infected in these studies. Interestingly, SARS-CoV-2 infection was reported to proceed in the following order from proximal to distal: ciliate, club, and thereafter AT2 cells [57, 58]. The regulation of metabolism is an important link in the infection process; glycometabolism and lipid metabolism are the 2 main pathways regulated after infection. Wang et al. detected the upregulation of glycometabolism on a human distal lung organoid model. qRT-PCR analysis showed that the expression of glycolysis-related genes, for instance, hexokinase 2, was upregulated, whereas the expression of oxidative phosphorylation-related genes, for instance, ubiquinone oxidoreductase subunit S6, was downregulated in SARS-CoV-2 infected cells. In conclusion, glycolysis appeared to be the main energy source for SARS-CoV-2 replication. The key enzymes involved in the downregulation of TAG biosynthesis might also influence SARS-CoV-2 replication, as the expression of the 2 metabolism-related receptors NR1H3 and NR1H4 was also shown to be downregulated [57-59]. A study on the mechanism underlying the invasion of SARS-CoV-2 detected the formation of a loop by the S1 and S2 domains that might facilitate viral entry. The loop, named after MBCS, facilitated TMPRSS2-mediated S protein cleavage in an airway organoid model. This process facilitated the binding of RBD on the S1 protein to ACE2 and promoted invasion. However, cell-cell fusion is known to be more common in viruses with MBCS than in viruses with mutant MBCS, because of the higher number of syncytia [60, 61]. To improve the SARS-CoV-2 infection ratio in AWO and to better model the human respiratory tract, Chiu et al. replaced the original buffer solution on the organoid surface, namely HEPES, with another buffer, PIPES, resulting in a weakly acidic PH, which facilitated viral infection. It is worth mentioning that the Omicron variant showed higher infectivity and replicative fitness in this model, which was in accordance with the characteristics of the mutants previously mentioned [53].

### 4.2. Gastrointestinal Organoids

In addition to respiratory symptoms, gastrointestinal symptoms are among the most common manifestations of COVID-19 [62]. A study of hospitalised patients in Italy showed that gastrointestinal symptoms included diarrhoea, anorexia, vomiting, and abdominal pain, of which diarrhoea was the most common [63]. Another study showed that SARS-CoV-2 RNA was detected in faecal samples, with the duration of positivity being even longer than that in respiratory tract samples [64]. However, viruses in the faeces were shown to be inactivated by the intestinal fluids, with the residual viruses exhibiting limited pathogenicity [65]. This suggested that the gastrointestinal tract might also be an important site of viral infection and that faeces constitute a potential source of infection. Therefore, it is necessary to research the relationship between the gastrointestinal tract and SARS-CoV-2 infection [66].

**Figure 3.**
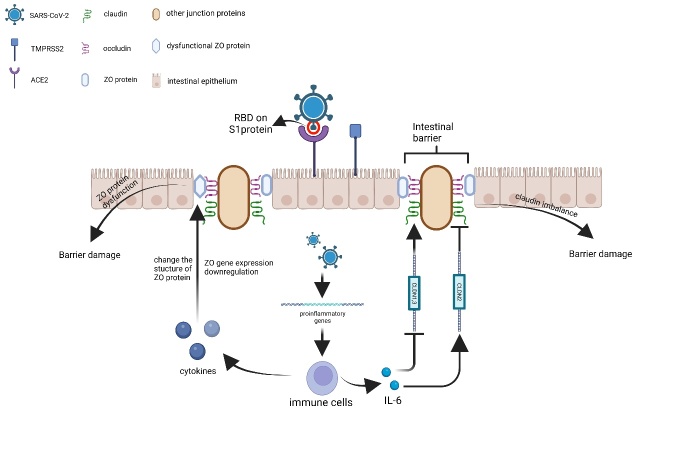
Inflammatory factors are the main reason for the SARS-CoV-2 infection-induced intestinal barrier damage. The expression of cell tight junction genes, mainly *ZO*, is downregulated and the structure is damaged by the increased levels of cytokines. ZO protein dysfunction is one of the reasons for barrier damage. Claudin depends on the expression of CLDN, which is influenced by IL-6; hence, the imbalanced expression of these proteins is another reason for barrier damage.

Individualised levels of ACE2 were retained in colonic and small intestine organoids, and in comparisons of the highest and lowest levels of expression of ACE2, a 5.9 times difference in the level of ACE2 resulted in a 423 times difference in viral load. Immunofluorescence of susceptible and resistant organoids also showed that the level of ACE2 staining was higher in susceptible cells. Therefore, individualised susceptibilities do exist and are mainly attributed to the levels of expression of ACE2 [37, 67]. The viral-induced damage in the intestinal barrier was studied in an iPSC-derived intestinal epithelium organoid. The downregulation in the expression of cell tight junction genes, such as ZO-3 CLDN1, which occurred following SARS-CoV-2 infection, was found to cause barrier damage (Fig. 3). Conversely, the expression of pro-inflammatory genes, namely CCL2, CCL3, IL-1b, and IL-6, was upregulated [68]. Moreover, a series of inflammatory reactions and injuries occur following viral entry in host cells. IFN plays an important role in infection and immune responses [69, 70]. However, the IFN response seems to be delayed in both proximal intestinal and colonic organoids; the response induced by a low-load viral infection was not robust, whereas IFN-related pathways were upregulated under high-load viral infection [71]. Both IFN I and IFN III can protect intestinal epithelial cells from viral infections but differ in their kinetics. IFN I acts more rapidly than IFN III because of the inherent differences in their signalling pathways, but not in their abundance [72]. A recent study showed that compared with IFN I, the IFN III response was more efficient and rapid in SARS-CoV-2 infection, in contrast to the results of previous studies. This might be explained by the virus-specific characteristics of IFNs [73]. Zeng et al. found a new inflammation pathway during the SARS-CoV-2 infection, which activated the ERK/VEGF pathway, increased vascular permeability, resulting in the leakage of inflammatory factors and the intensification of inflammatory reactions. These findings provided a new treatment target for the control of inflammation in patients with COVID-19 [74]. In contrast to intestinal organoids, the inflammatory response in adult gastric organoids was demonstrated to be moderate, and was associated with low levels of viral replication and proliferation; instead, viruses seem to be active in late foetal and paediatric organoids [75].

Researchers have made several efforts to improve the original organoid model. Zhao et al. removed R-spondin and Noggin from the traditional media, leading to improved differentiation. The organoids cultured in this medium consisted of Paneth, goblet, and enteroendocrine cells, more closely resembling the human intestinal tract [76, 77]. Qu et al. established an organoid model in a novel medium called the 8C medium, consisting of LDN193189, GSK-3 inhibitor XV, pexmetinib, VPA, EPZ6438, EGF, R-Spondin 1-conditioned medium, and bFGF. This complementation of the traditional media (R-spondin, EGF, and Noggin) facilitated faster and stronger growth and proliferation than before. In addition, the novel model exhibited a more complex crypt-villus structure and might thus be used in the future in intestinal regeneration research for the treatment of COVID-19-associated intestinal symptoms [78]. Nikolaev et al. developed a tubular intestinal organoid to maintain long-term homeostasis. The organoid was based on a scaffold composed of a mixture of type-I collagen, which allowed fluid perfusion and the clearance of necrosis and cast-off cells, prolonging its lifespan. Moreover, it showed a permissive connection with external factors, including the medium, drugs, and microorganisms [79].

### 4.3. Hepatocholangial Organoids

**Figure 4.**
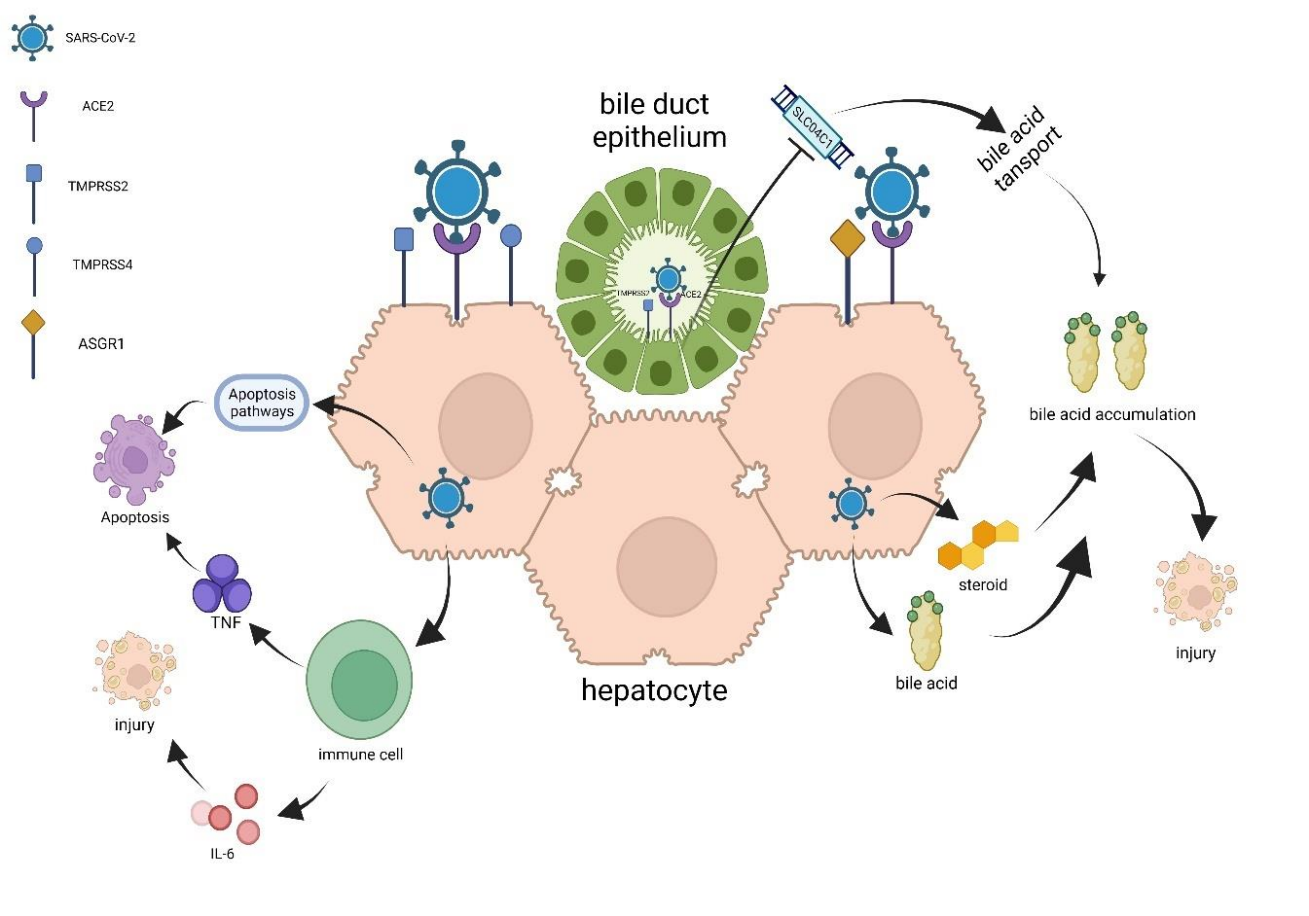
Hepatocyte injury is mainly caused by the inflammatory response and accumulation of bile acids. ASGR1 and TMPRSS4 are both important assistive receptors for SARS-CoV-2 invasion. Infection of the bile duct epithelium decreases the expression of the bile acid transport gene SLCO4C1 and causes the accumulation of bile acids, finally leading to hepatocyte injury. In addition, IL-6 directly damages hepatocytes, and together with the activation of pro-apoptotic pathways promotes hepatocyte injury.

The autopsy of patients with COVID-19 revealed liver steatosis, hepatitis, and abnormal enzymes, as well as viral RNA in liver tissues [80]. Liver damage was pronounced in patients with COVID-19; however, we do not know if the virus infects hepatocytes directly or harms the liver through a systemic inflammatory response. Richards et al. established pluripotent stem cell-derived liver organoids, which revealed that inflammatory pathways, including IL-6 secretion and IFN signalling, were the main cause of hepatocyte damage [81, 82]. Lui et al. studied intrahepatic bile duct organoids and detected the expression of ACE2 and TMPRSS2, indicating that this organoid was permissive to SARS-CoV-2 [83]. Two more studies conducted on similar organoids showed that the observed liver injury might be attributed to the accumulation of bile acid caused by the downregulation of the bile acid transporter gene SLCO4C1, and biliary epithelial barrier damage. (Fig. 4). Gene sequencing after SARS-CoV-2 infection showed the upregulation of pro-inflammatory pathways, including the NF-kB pathway, and the strong induction of cytokine production. The upregulation of TNF and apoptosis pathways was also observed in liver and biliary organoid models. Upregulation of these pathways might induce the death of hepatocytes and bile duct cells [84, 85].

### 4.4. Urinary System Organoids

**Figure 5.**
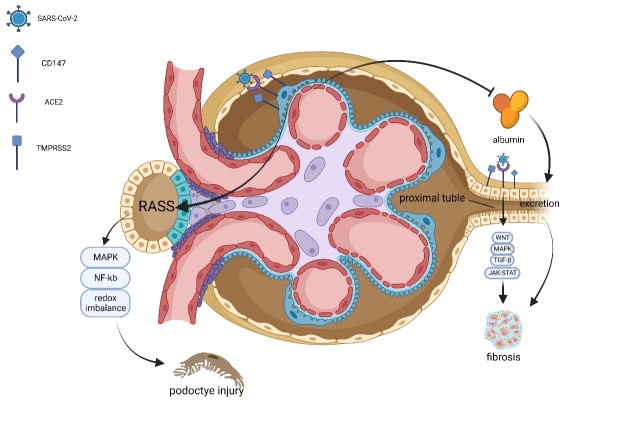
ACE2 activates the RASS system leading to tissue damage, whereas it decreases the excretion of albumin, protecting the kidney. Except for TMPRSS2, CD147 is another important assistive receptor for SARS-CoV-2 invasion. Podocytes and tubular epithelial cells are the main target cells of SARS-CoV-2 infection. The deposition of collagen I and upregulation of profibrotic pathways cause tubular epithelium cells fibrosis and the activation of NF-kb and other pathways, resulting in podocyte injury.

Kidney injury, including acute kidney injury (AKI), is common in patients with COVID-19, with abnormal proteinuria appearing in >40 % of hospitalised patients [86]. The underlying pathogenesis is complicated, involving both direct and indirect factors. ACE2 remains important for viral invasion, with some proteases expressed in the kidney also participating in this process [87]. Interestingly, the role of ACE2 was shown to be two-sided in kidney injury; it activated RASS and generated Ang II resulting in damage, whereas, at the same time, it decreased albumin excretion, protecting the kidney. An imbalance in the expression of ACE2 was shown to promote kidney injury [88]. Post-mortem histopathological analyses indicated that podocytes and tubular epithelium, which are the main targets of direct viral infection, were the main sites expressing ACE2. Although TMPRSS2 is not as important as in other tissues, CD147 was reported to assist in the invasion of SARS-CoV-2 [89]. Using iPSC-derived kidney organoids, Jansen et al. showed that podocytes and proximal tubular epithelial cells were infected with SARS-CoV-2, leading to increased inflammation and collagen I deposition, upregulating pro-fibrotic signalling pathways (WNT, EGFR, FGF, and JAK-STAT), and resulting in kidney fibrosis [90] (Fig. 5). Xia yet al. established a model using both conditional reprogramming and organoid culture. This clever combination achieved the maximum approximation of the human kidney structure, reserving specific transporters, such as SLC34A3, repairing damaged DNA, and realising the long-term stability of the organoid model [91]. Garreta et al. established diabetic kidney organoids using hPSCs and alternating high and low glucose concentrations. The high glucose conditions induced a high expression of ACE2, followed by increased levels of viral mRNA. Moreover, aerobic oxidation caused higher susceptibility, whereas OXPHOS inhibited viral replication [92]. These studies highlighted the availability of certain new targets for inhibiting infection and kidney injury. The feasibility of metabolic regulation and use of pro-fibrotic signalling pathway inhibitors should be evaluated in future research.

### 4.5. Nervous System Organoids

**Figure 6.**
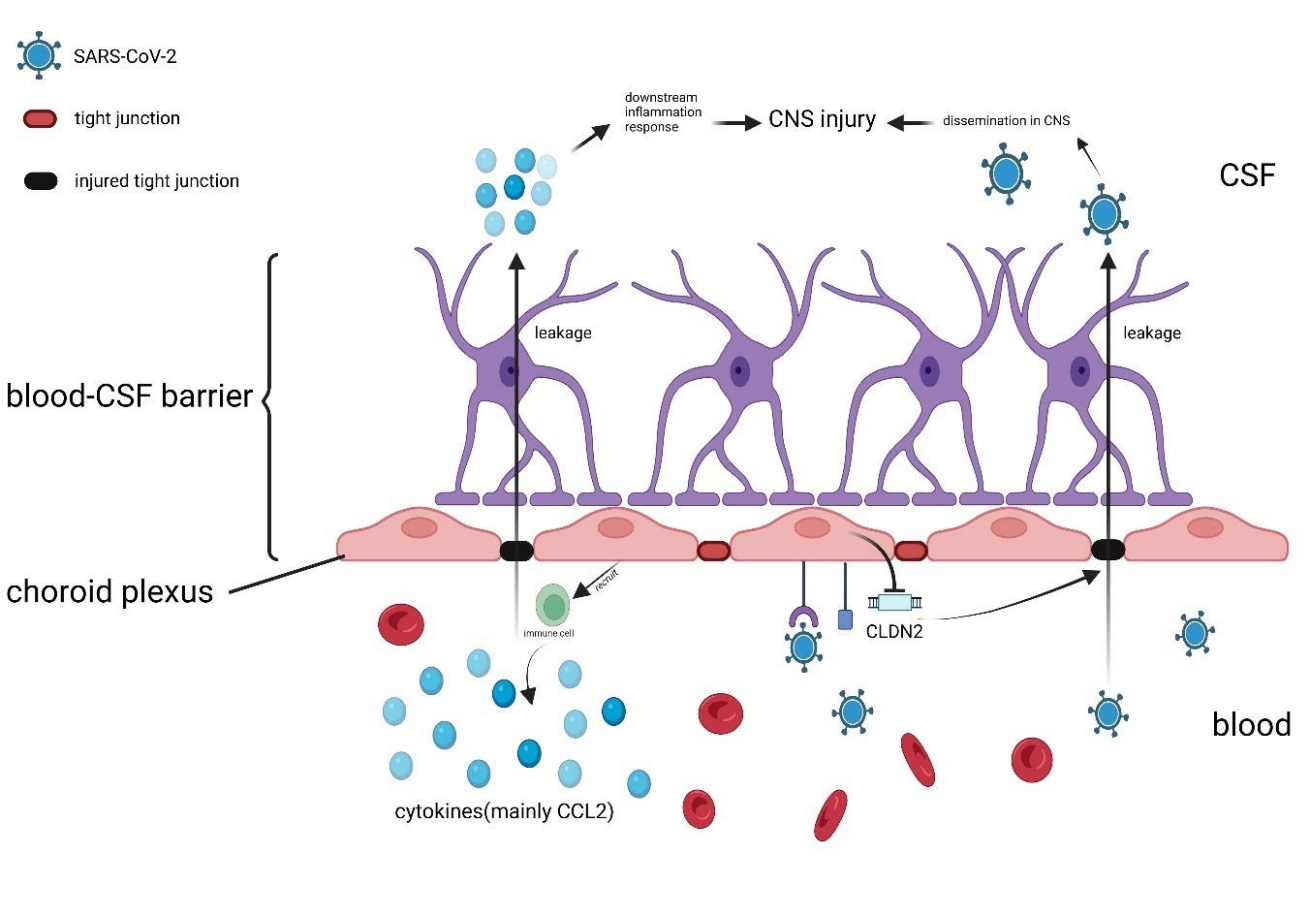
Blood-CSF barrier leakage is the main reason for central nervous system (CNS) injury. Downregulation of the cell tight junction gene CLDN2 causes damage in the tight junction between choroid epithelium cells. Increased levels of cytokines and viral particles in peripheral blood leakage might induce downstream inflammatory responses and viral dissemination in CNS.

Various studies have mentioned the incidence of neurological complications, such as encephalitis, encephalopathy, and Guillain-Barré syndrome, in patients with COVID-19 [93]. However, the mechanisms of viral invasion in the central nervous system remain unclear. The choroid is an important tissue for the formation of cerebrospinal fluid (CSF); a high expression of ACE2 has been observed in the choroid, which might lead to the virus spreading through the CSF [94]. Two studies on choroid organoids revealed a partial mechanism of infection involving transcriptional disorders and upregulation of the pro-inflammatory pathway, especially CCL2, both of which induced a severe inflammatory response, in turn causing cell injury. Downregulation of the cell junction gene *CLDN2* might result in damage to the tight junctions between choroid epithelium cells, hence disrupting the blood-brain barrier (Fig. 6). CSF leakage was shown to generate a violent inflammatory response that led to the development of neuritis [95, 96]. Jing-Han et al. also indicated that immune and inflammatory responses were the main causes of cell death [97]. In an autopsy of a patient with COVID-19, abundant antibodies were detected in the CSF, further verifying the above viewpoint [98]. Nonetheless, studies on neurotropism have been inconsistent; one study showed that the basal level of expression of ACE2 in neurons was adequate for SARS-CoV-2 infection, and correlated with unexplained abnormal levels of phosphorylation of the Tau protein, which was suggested to generate downstream effects that induced stress and cell death [99]. Another study using iPSC-derived human dorsal forebrain organoids also verified that adult neurons express ACE2 [100]. However, the outcomes of two studies on neural progenitor cells were adverse; Zhang et al. reported that ACE2, TMPRSS2, and furin were expressed in neural progenitor cells, inducing cytotoxicity, but without pro-inflammatory conditions or upregulation of the IFN pathway, indicating a different infection model of neural progenitor cells [6]. In another study on brain organoids, neural progenitor cells and neurons showed the lowest susceptibility to SARS-CoV-2 than previously reported [97]. An organoid model derived from embryonic stem cells revealed that gilia cells, especially astrocytes, were the primary targets of SARS-CoV-2, whereas neurons seemed to be almost non-susceptible to infection [101]. These inconsistent outcomes might be due to the usage of different cell sources and culture environments; thus, further research is needed to better explore SARS-CoV-2 neurotropism.

### 4.6. Cardiovascular System Organoids

Acute myocardial injury and arrhythmia have also been observed as complications of hospitalised patients with COVID-19, with those with basal heart diseases showing a higher risk of adverse outcomes and prognosis. Cytokine storms have been suggested to be the main causes of cardiac injury, and an increase in the levels of CRP in direct proportion to increasing serum levels of TnT further validated this viewpoint [102, 103]. An experiment using hPSC-derived human cardiac organoids showed that increasing levels of IFN-g, interleukin-1b, and TNF resulted in myocardial dysfunction, whereas BETi inhibited SARS-CoV-2 transcription and any viral-induced cytokine storms, thus relieving myocardial injury [104, 105]. In addition to the indirect effects, direct infection was also observed in hPSC-derived cardiomyocytes and smooth muscle cells. Although the virus was shown to infect cardiomyocytes instead of smooth muscle cells; however, a decreased expression of ACE2 on the cardiomyocyte surface resulted in the cooperation of virus-cardiomyocyte membrane fusion and fusion within cardiomyocytes, ultimately causing both electrical and mechanical dysfunction [106]. Another study on iPSC-derived cardiac cells attempted to reveal the mechanism of myocardial injury. Myofiber fractures are the main manifestations of myocardial injury, mainly attributed to the function of the Papin-like protease, while also assisted by the inhibition of the ubiquitin-proteasome system. Lack of nuclear staining was also observed in cardiac muscle cells, which might be explained by the disruption of the cytoskeleton [107].

As the first functional organ during the developmental process, reprogramming a functional cardiac model with appropriate morphology and electrophysiology function is difficult. The chambered cardiac organoid is an in vitro cardiac model with chambers that closely resembles human physiology. In order to establish correct cardiac organoids, researchers have investigated the key points in the culture process. Hofbauer et al. constructed a chambered cardiac organoid model derived from mesoderm cells and found that high levels of Wnt signals were especially important for chamber formation, as well as Hand 1 was also essential. The Wnt-BMP signalling axis was reported to be key for self-organizing, also regulating the chamber formation process [108, 109]. Ho et al. established a chambered cardiac organoid derived from pluripotent stem cells and demonstrated that a suitable ratio of cardiovascular progenitors and cardiac muscle cells was correlated with chamber formation and contractile function [110]. In another study, VEGF was added during the culture process to promote angiogenesis on the basis of chamber formation. The vascularized cardiac organoid might more accurately simulate the heart damage caused by the increased levels of cytokines or inflammatory factors in peripheral blood [111]. These studies have provided novel insights on the means by which to establish a cardiac organoid model closer to human physiology. Cardiac organoids will become an excellent model for future studies on SARS-CoV-2 infection and heart damage in patients with COVID-19.

### 4.7. Vascular Organoids

Damage to the vascular system is mainly reflected via endothelial permeability and protein-protein interactions that suggest the viral-induced damage of endothelial proteins, leading to barrier damage. Certain proteins, including nsp2, nsp5, c145, and nsp7, are essential for transcription of SARS-CoV-2 genes, and damage the catenin and cadherin of the tight and adherent junctions of vascular endothelial cells. The stabilisation of vascular permeability relies on the cooperative function of endothelial cells and pericytes. Research regarding vascular organoid models indicated that pericytes were the preferential targets of SARS-CoV-2, in turn damaging the network of pericytes and endothelial cells and resulting in increased permeability [112, 113].

### 4.8. Other System Organoids

### 4.8.1. Ocular Organoids

Ocular manifestations may not be obvious in the initial stages of COVID-19; however, conjunctivitis is the most common ocular symptom in the progressing disease[114]. Both ACE2 and TMPRSS2 were shown to be highly expressed in the superficial limbal, corneal, and conjunctival epithelium, particularly in the limbal, and were associated with high expression of TMPRSS4 [115, 116]. Direct infection of the ocular surface might result in retinal or even central nervous system infection. In an iPSC-derived retinal organoid, ACE2 and TMPRSS2 were co-expressed in the retinal epithelium. Interestingly, SARS-CoV-2 infection enhanced the inflammatory response, especially the level of IL-33, in organoids, potentially promoting the expression of ACE2 and TMPRSS2, while simultaneously promoting injury [117, 118].

### 4.8.2. Tonsil Organoids

The tonsil epithelium also expresses ACE2 and TMPRSS2, suggesting that tonsils are potential targets for SARS-CoV-2 infection. Kim et al. established a human tonsil organoid and showed that tonsils were the main target for primary viral infections; the original immune response was suppressed as a result of the virus inhibiting the proinflammatory cytokine pathways, resulting in viral survival and reserves [119]. Importantly, tonsil organoids might be an efficient model for SARS-CoV-2 vaccine testing because of their immunoreactivity. This model has been successfully applied for studying the immune response, following vaccine administration. Significant activation of CD8^+^ T-cells and differentiation of plasmablast was observed in the group injected with the candidate vaccine (compared with the control group). In addition, specific IgG and IgA antibodies against SARS-CoV-2 were also detected in the tonsil organoid model. Both findings indicated its potential use as an effective model in SARS-CoV-2 vaccine studies. Individualised immune responses can be recorded in future studies that would facilitate the development of a vaccine with extensive immunoreactivity [120].

### 4.8.3. Capillary Organoids

Capillary organoids are permissive to SARS-CoV-2 infections, and tests using this model indicated that infection of the blood vessels was essential for local tissue infection. The virion was shown to be able to cross the vascular wall and enter the endothelial cells; thereafter, the virus replicated and released its progeny, finally leading to dissemination. This study shed new light on the mechanism by which the virus spreads throughout the body, leading to multiple organ infections [121].

### 4.8.4. Salivary Organoids

Saliva is a potential source of SARS-CoV-2 transmission. In order to reveal the interpersonal transmission mechanism, Junichi et al. established a salivary gland organoid derived from hiPSCs, which perfectly simulated the morphology, function, gene expression, and development of human salivary glands. In this model, virus-like particles were detected in ductal luminal cells, while SARS-CoV-2 genomic genes were detected in the supernatant and cell lysates; both results recapitulated SARS-CoV-2 infection. In addition, ACE2 and TMPRSS2 were strongly expressed, and other potential viral entrance points, such as TMPRSS4, CD147, and furin, were also broadly expressed on organoid cells. In brief, salivary gland organoids are an excellent in vitro model for studying the interpersonal transmission of SARS-CoV-2 [122].

### 4.8.5. Skin Organoids

Skin injury, such as acral areas of erythema, acral areas with erythematous rash and widespread urticaria, is another clinical manifestation in patients with COVID-19. A recent study also indicated hair loss as another manifestation. Ma et al. established a hiPSC-derived skin organoid with a typical stratified epithelium structure and mature epithelium markers and challenged this model with SARS-CoV-2 to reveal the mechanism of skin injury. They found that hair follicles were targets of infection, which explained the observed hair loss. The generation of proteins related with basal layer stem cells and basement membrane component in hair follicles was downregulated during infection. Neurons in skin tissue were also targeted by SARS-CoV-2, in a process similar to that occurring in the brain. In summary, both hair follicle and neuron injury were highlighted as potent reasons for skin damage [123].

Different tissue organoid models have increased our understanding of the mechanisms involved in the pathogenesis of COVID-19 in the human body. According to current studies, studies using organoid models have come to the same or similar conclusions as those obtained from autopsies, verifying their feasibility and further expanding the application of organoid models for the study of the mechanism of infection. However, the investigation of the mechanisms related to the infection of human organs by SARS-CoV-2 should not be limited to the organoids described above; for example, until now, no studies have been performed on genital system organoids, although the testis is a target of SARS-CoV-2. Immune or inflammatory responses caused by viral infections might result in orchitis, influencing male reproduction [124]. Further studies are needed regarding the mechanism of infection, and organoids will serve as an excellent experimental model. In addition, various studies using organoids have reached different conclusions. For example, differences were observed in the tropism of SARS-CoV-2 in nervous system cells after challenging the organoids with the virus. In addition, the effects of each type of IFN on SARS-CoV-2 infection, regulation of ISGs and IFNs against the virus, and opinions on these processes have been inconsistent. Further studies are required to clarify these outcomes. However, irrespective of the limitations of these studies, organoids are expected to perform well in future studies.

## 5. Organoids Remodel the Research on Drug Screening and Vaccine Research against SARS-CoV-2 Viruses

**Figure 7.**
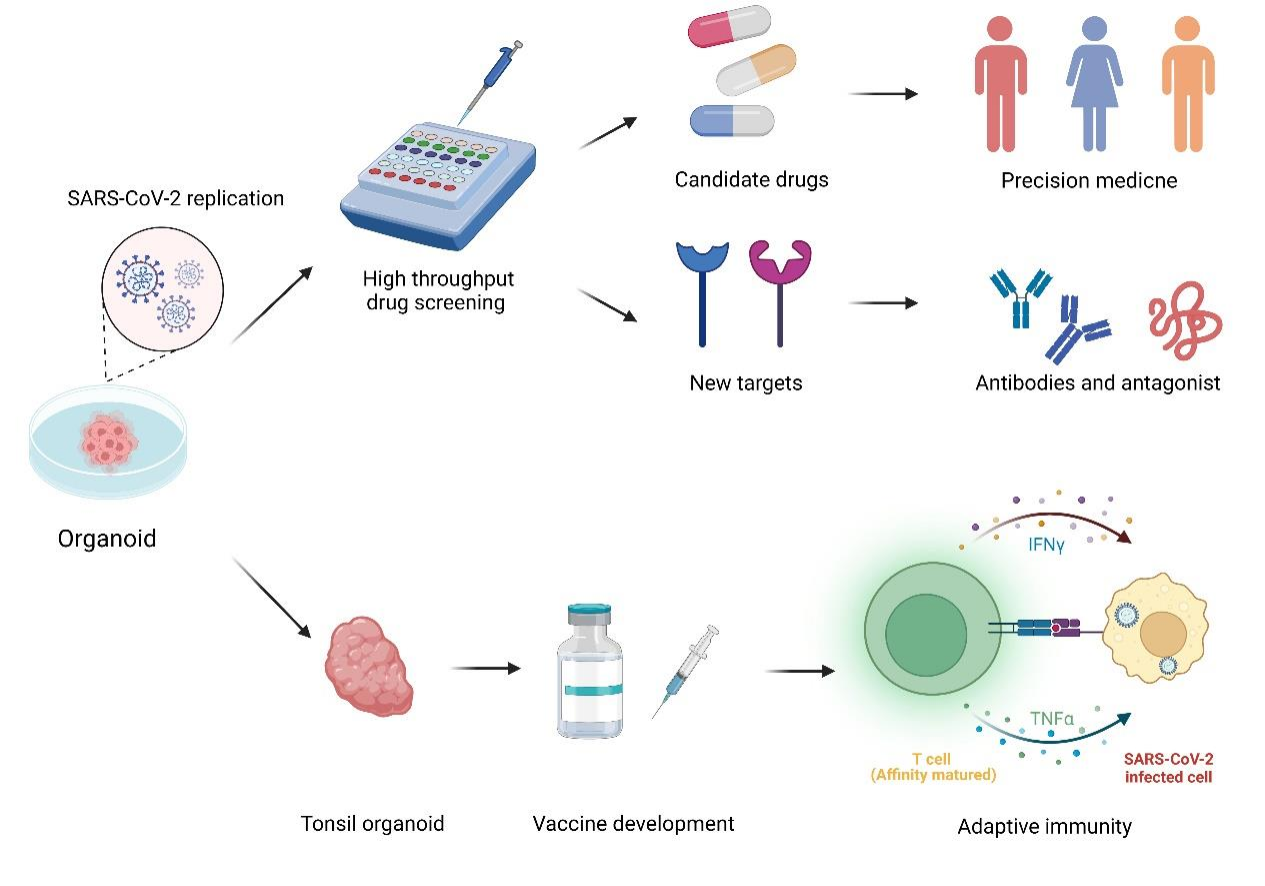
Drug screening using organoids has the advantage of being fast, having high throughput, and strong clinical relevance. Individualized therapies can be developed through the establishment of individualized organoid models, which can also assist researchers in identifying new targets for improved therapeutic approaches. SARS-CoV-2 infected tonsil organoids exhibited matured immunoreactions, suggesting their potential application for future vaccine research and development.

To control outbreaks of COVID-19, drug screening and vaccine research are essential. While the development of clinical drug tests is limited by the number of experimental subjects, tests on animals cannot reflect the real response in vivo. Drug screening on organoids has the advantage of being fast, having high throughput, and strong clinical relevance; hence, organoids are supposed to be excellent models for drug screening (Fig. 7).

### 5.1. Respiratory System

Owing to their observed efficiency in both lung and colon organoids, mycophenolic acid (MPA), imatinib, and quinacrine dihydrochloride (QNHC) were selected as potential drug candidates [125]. Katsura et al. developed a lung stem cell-based alveolosphere model and found that pre-treatment with IFN prevented SARS-CoV-2 infection in alveolospheres; the study also indicated that further research should be conducted to determine whether IFN can prevent infection and viral spread among humans [52]. However, in another study, opposing results indicated that IFN upregulated the expression of ACE2 and thus promoted SARS-CoV-2 infection. We speculated that IFN might play a two-tier role in the infection of lungs by SARS-CoV-2 [126]. DPP-4 is a transmembrane glycoprotein that might also act as a potent receptor of SARS-CoV-2. A study assessed a new antibody (Fab15033-7) targeting RBD and a new peptide (DPP-4 270295) isogenous with the DPP-4 receptor on host cells in organoid models. The antibody blocked the binding of the spike protein to ACE2 receptors on host cells, whereas the peptide inhibited viral binding to the DPP-4 receptor [127]. A number of antineoplastic drugs were evaluated by Ye and his colleagues, who indicated that bevacizumab, thalidomide, and imatinib might alleviate symptoms in patients with severe COVID-19 [126]. Remdesivir was also considered to be more efficient than a placebo for the treatment of hospitalised patients with COVID-19; it prevented the progression of respiratory disease, leading to faster recovery [128]. However, recent studies showed that remdesivir was only effective for specific low-risk patients in recovery, whereas it seemed to be inefficient in high-risk patients [129, 130]. Human soluble ACE2 has also been considered a synergist of remdesivir, and a combination of these 2 drugs might enhance the efficiency of remdesivir [131]. In addition, remdesivir promoted the proliferation of club cells on mouse lung organoids; these cells are progenitor cells of the respiratory tract. We can therefore assume that remdesivir can repair the injured epithelium, while lopinavir, ribavirin, and ritonavir also affect the regeneration of the epithelium [132]. Atorvastatin, a lipid-regulating drug, downregulated the expression of genes related to IFN, chemokines, and complement activation, and inhibited SARS-CoV-2 entry into a lung organoid model. Thus, atorvastatin might relieve the inflammatory response and injury in vivo [133]. GW6471, a PPARα antagonist, downregulated the HIF signalling pathway in glycolysis and inhibited the glycolysis axis, which provides the virus energy for survival. Fatty acids are significant for viral replication; however, their synthesis was also inhibited due to the lack of pyruvic acid generated by glycolysis. Thus, GW6471 inhibited SARS-CoV-2 infections by modulating the energy metabolism as verified in hPSC-derived AO and CO models [59].

### 5.2. Digestive System

Remdesivir has also been demonstrated to be effective in the intestinal tract. Krüger et al. established an iPSC-derived human intestinal organoid and assessed the administration of remdesivir, EK1 (a peptide pan-coronavirus fusion inhibitor), and famotidine in this organoid. They showed that both remdesivir and EK1 were efficient in preventing SARS-CoV-2 infection, whereas famotidine did not decrease the rate of viral infectivity [134] Remdesivir was also reported to repair the injured intestinal barrier and increase transepithelial electrical resistance (TEEN); whereas, SARS-CoV-2 infection decreases TEEN and promotes the expression of inflammatory-related genes. This finding was similar to that of a study conducted on lung organoids; therefore, remdesivir appears to exhibit robust repair abilities in injured organs [68]. However, another study reported that famotidine decreased the mortality and incubation rates, due to exacerbation in critical patients [135]. A number of interesting studies have provided novel methods for the treatment of patients with COVID-19. In particular, a study demonstrated that human milk inhibited SARS-CoV-2 infections by suppressing the ATP1A1-mediated endocytic virus. This study was conducted in foetal enteroids, and hence it is not clear whether human milk is still effective in the adult intestine. Further studies are needed to verify this finding [136, 137]. In a case report, enema treatments with traditional Chinese medicine were shown to inhibit the spread of SARS-CoV-2 in the intestine, curing intestinal symptoms and preventing faecal-oral transmission [138]. In conclusion, only remdesivir has been demonstrated to be effective against intestinal symptoms and in the control of SARS-CoV-2 infection; famotidine might benefit critical patients, whereas more evidence is required for the effectiveness of other novel drugs.

### 5.3. Urinary System

The RAS inhibitor losartan has been shown to diminish the generation of Ang II, while simultaneously inhibiting SARS-CoV-2 infection. In an iPSC-derived human kidney organoid, losartan upregulated the expression of IFITM1 and BST2, inhibited the internalisation of ACE2, and prevented SARS-CoV-2 infection [139]. The JAK-STAT signalling pathway might also mediate COVID-19-induced cytokine storms, leading to the upregulation of *APOL1*. This hypothesis was evaluated in an iPSC-derived kidney organoid cultured with COVID-19-induced cytokines, in which it was shown that SARS-CoV-2 ultimately caused organoid podocyte injury. The JAK inhibitor, baricitinib, was applied to this organoid to block cytokine-mediated podocyte injury, achieving remarkable results [140]. However, whether baricitinib blocked the pro-fibrotic signalling pathway and inhibited kidney fibrosis remains unknown. The use of remdesivir is also limited because of its hepatic and renal toxicity. Thus, the dosage should be noted when used, especially in patients with kidney disease [141]. Human soluble ACE2 has been evaluated in human kidney organoids in 3 studies. In one of them, it enhanced the effect of remdesivir and independently inhibited infection. A low-dose combination of remdesivir and human soluble ACE2 diminished toxicity and showed additive effects on human kidney organoids and Vero E6 cells [131]. Wysocki et al. tested a new soluble ACE2, with 618 amino acids, which was fused with the albumin binding domain. They assessed it in human kidney organoids, in which the beneficial effect lasted longer; hence, this variant provided a convenient dosage regimen with improved efficacy [142].

### 5.4. Vaccine research

Vaccine injections have shown to be an effective method for neutralising the virus and preventing poor prognosis in infected patients. However, there is still a need for a broadly immune suitable vaccine against the various variants, such as Omicron and Delta. The vaccines that are currently clinically used cannot keep generating high-level neutralising antibodies against different mutants [129, 143, 144]. Therefore, vaccine development is ongoing. The tonsil organoid model, which was shown to generate an immune response following challenging with a live attenuated influenza vaccine, provided a new method for vaccine research. The immune response process included B-cell maturation, plasmacyte generation, activation of CD4^+^ and CD8^+^ T-cells, and affinity maturation. Similar outcomes were observed after administration of the SARS-CoV-2 vaccine, including plasma blast differentiation and activation of CD8^+^ T-cells; this indicated that the in vivo immune response can be excellently mimicked in organoid models [120]. Owing to the limitations of clinical trials and animal models, organoids will be an outstanding model for future vaccine research and development.

The FDA implemented policies on alternative methods, which stipulate that experiments on animal models should follow the 3R rules. This policy calls for the use of alternative methods and a reduction in the use of live animals; organoid models and organ-on-a-chip are included among these alternative methods.(www.fda.gov/science-research/about-science-research-fda/advancing-alternative-methods-fda) Another policy suggested that alternative systems, including organoids, should be applied for determining the potential toxicity of chemicals and various responses to toxicological effects in the population.(www.fda.gov/about-fda/domestic-mous/mou-225-22-005)Thus, organoids have been considered as the most suitable model by the FDA for drug screening; additionally, hazards to experimental animals will decrease once alternative methods are implemented.

In summary, in the treatment of COVID-19, none of the drugs studied has been determined to be effective enough to date, indicating the combination of drugs as a new potential solution. A considerable number of drugs are still in the theoretical test stage and have only been shown to be effective in in vitro cell experiments. Although several clinical trials have been conducted, the development of new drugs remains unclear. In addition, organoids might be an effective model for revealing the immune response after vaccine administration in future studies exploring the preventive effect of vaccines.

## 6. Conclusion, limitations, and perspectives

The COVID-19 pandemic has claimed millions of lives, highlighting the urgent requirement of extensive research to gain insights into the mechanisms and effects of SARS-CoV-2 infection. As emerging 3D models, organoids provide researchers with an experimental platform that remarkably resembles human physiology. Moreover, organoids have been widely used in the study of infectious diseases, thereby verifying their feasibility and reliability. In this review, we summarised the applications of organoids in the research of SARS-CoV-2 pathogenesis and drug screening and outlined the main points in Table 1. We observed that cytokine storm-induced organ injury was a common outcome in almost every model tested and verified that the interaction of SARS-CoV-2 with ACE2 and TMPRSS2 plays an important role in the process of direct infection. Following the binding of RBD of the S protein to ACE2 on receptor cells a number of downstream responses occurred, including the excessive activation of inflammation and dysregulation of gene transcription and translation, all of which contributed to the damage of related organs. None of the drugs mentioned in this review has been shown to be completely effective against COVID-19; remdesivir seemed to be the most reliable drug, however, its use is limited owing to its side-effects, such as liver and renal toxicity. A combination of drugs might reduce toxicity and increase efficiency, and some newly found key links in the infection process might be the focus of future research. However, the use of organoids is still associated with certain limitations.

## 1. Heterogeneity and repeatability

Owing to the intrinsic cell fate orientation and self-organisation of stem cell-derived organoids, organoids are generally heterogeneous in size, shape, and cellular composition. This heterogeneity can occur regardless of whether organoids are derived from different patients, established in different laboratories, or obtained from the same stem cells. This lack of standardisation creates enormous difficulties and reduces the use of organoids in disease modelling and drug discovery. Therefore, there is a need to further standardise and automate these procedures using culture media and starting cell types, to reduce variability and improve system repeatability. Microfluidic technology ensures the highest degree of consistency in the culture environment; it better models the dynamic process of organ development and provides researchers with more precise control over the direction of differentiation.

**Table 1:** Various types of organoids and their corresponding characteristics.

Type	Cell source	Cell containing	Main target	Key factors	Drug screening	Refs.
Nasal organoid	Stem cell from turbinal tissue	Pseudostratified cell Ciliated cell Goblet cell Club cell Basal cell	Ciliated cell	IL-6 IL-8 VEGF		[43]
Bronchial organoid	Adult stem cell	Pseudostratified cell Ciliated cell	Ciliated cell	CXCL8 CXCL10 EGF		[47, 48]
Alveolar organoid/ Alveolosphere	hPSC iPSC Adult stem cell	AT1 & AT2 cell Club cell	AT2 cell	CCL5 IFN TNFα IL-6 IL-8 NF-KB pathway	Remdesivir Nelfinavir Lopinavir MPA QNHC Imatinib	[50, 51, 125]
Airway organoid	ASCs isolated fromsurgically distal lung tissue hESC hPSC iPSC	Cells in both AO and BO	Ciliated cell Club cell AT2 cell	Glycometabolism axis HIF pathway MBCS loop	GW6471 Fab15033-7 DPP4270295 Atorvastatin	[57, 59, 60, 127, 133]
Intestinal organoid	iPSC hPSC ASC Primary gut epithelium stem cell			ZO-3 CLDN1 CCL 2, CCL3, IL-1b, IL-6	Remdesivir EK1 Human milk	[68, 134, 136]
Liver organoid	Liver bile duct-derived progenitor cells hPSC	Hepatocyte	Hepatocyte	IL-6, IFN		[81, 83]
Hepatobiliary organoid		Cholangiocyte Hepatocyte	Cholangiocyte Hepatocyte	SLCO4C1 TNF CD40 pathway		[84, 85]
Kidney organoid	hPSC iPSC		Podocytes tubular epithelium cells	OXPHOS RASS CD147 JAK-STAT pathway	Baricitinib Losartan Human soluble ACE2 Remdesivir	[90, 91, 131, 139-141]
Choroid organoid	hPSC	Choroid cell	Choroid cell	CCL2 CLDN2		[95, 96]
Brain organoid	ESC iPSC hPSC		Neuron Progenitor cell Gilia cell	Tau protein phosphorylation		[6, 97, 98, 100, 101]
Cardiac organoid	hPSC iPSC	Myocardial cell Smooth muscle cell	Myocardial cell	IFN-g, interleukin-1b TNF Papin-like protease		[104, 106, 107]
Vessel organoid	iPSC		Pericytes Endothelial cell	Catenin Cadherin		[113]
Retinal organoid	iPSC		Retinal epithelium Retinal ganglion cell	IL-33		[117, 118]
Tonsil organoid		Tonsil epithelium cell	Tonsil epithelium cell	ANXA9 LOR ATP6V1C2 SPTLC3 mRNA	An excellent model for vaccine tests	[119, 120]

## 2. Influence of Matrigel

In general, a prepared single-cell suspension of stem cells is added to an extracellular matrix (ECM) hydrogel to establish an organoid culture system. A drawback of organoid studies is the reliance on animal-derived Matrigel, most of which are not defined and can thus introduce differences into the organoid cultures, due to batch-to-batch variability. Local variations in the mechanical properties of Matrigel preparations can also lead to organoid variability and poor reproducibility. Most organoid models derived from PSCs or APCs require the use of exogenous artificial ECM, which greatly limits their clinical use and organoid transplantation in humans. In addition, the inclusion of Matrigel in culture systems increases the difficulty of organoid delivery, genetic manipulation, genome-wide genetic screening, and high-throughput drug screening. A type of de-cellularized organoid can solve this problem; the defined Matrigel will diminish the batch-to-batch variability and enhance the repeatability of organoid models. Another artificial synthetic PEG hydrogel can also be applied in organoid cultures, however, this Matrigel might still cause problems in organoid transplantation. Interestingly, liver and lung organoid models cultured on the above 2 Matrigel types have shown better consistency, overcoming the limitations of traditional Matrigel.

## 3. Lack of vascularisation structure

Blood vessels contribute to nutrient supply and differentiation. The lack of blood vessels leads to central necrosis of the brain organoids, further interfering with their normal development and neuronal migration routes. Although co-culture with vascular stem cells provided some solutions to the above problems, problems associated with nutrient supply and the real blood microenvironment still persist. Endothelial and mesenchymal stem cells co-cultured with organoid models successfully generated vascularised organoids. Transplanting vascularised organoids into mice resulted in the connection of the organoid with the host bloodstream [145]. Importantly, a vascularised organoid model is more complete and reveals better cell-cell communication; in addition, the vascular network provides possibilities for organoid transplantation.

## 4. Lack of immune cells

The progress in immunotherapy has greatly promoted tumour research. However, owing to the problems of species specificity, the complexity of humanised systems, and partial or ineffective reconstruction of the immune system, immuno-tumour models are facing great challenges. There is an urgent need for in vitro models that can be used to individually verify efficacy. The emergence of organoids has provided a new opportunity for tumour immunotherapy. However, the lack of immune cells in the existing organoids limits their development. This limitation can be overcome by the co-culture of immune cells with organoids, in a microfluidic device. A co-culture of liver organoids with CD8^+^ T-cells was challenged with HCVs; the co-cultured CD8^+^ T-cells successfully identified and responded to HCVs. In addition, this method can be applied not only for T-cells but also other immune cells, revealing the potency of co-cultured organoids in immune response studies [146].

## 5. Structural and functional limitations

Typically, liver organoids lack specific cell types found in the native tissues and cannot generalise the full functional repertoire of the target organ. For example, most liver organoid models contain only 1 or 2 cell types and lack a functional bile duct network. Even the best multicellular liver organoid models lack a normal liver-partitioning pattern. Moreover, because of the epigenetic barrier encountered during hPSC differentiation into mature cells and the limited period of organoid cultures, hPSC-derived organoids generally fail to functionally mature beyond the foetal stage.

## 6. Low degree of organ systematisation

Many diseases develop and progress by the joint response/action of multiple organ systems. Although the traditional single organoid can simulate the environment of a single organ in vivo, more realistically, there are still some gaps in organoid usage for diseases involving multiple systems. Therefore, the construction of multisystem synergistic organoids is the new research direction for addressing this problem.

Multi-organoid-on-chips connect multiple organs using microfluids, thus enabling organoid systematisation and complexation. This model has been used in pharmacokinetic and toxicokinetic drug studies. Various combinations of organoids have been tested on chips, including intestine, lung, liver, and even tumours, resulting in excellent outcomes [147]. Therefore, we might utilise this model to study the systematic responses in patients with COVID-19 and better understand the mechanism of SARS-CoV-2 infection.

Previous reviews have summarised current organoid applications in relation to COVID-19 and other infectious diseases. The data show that 3D organoid models may have substantial similarity with human physiology, and consequently, they have been used in a wide variety of research such as studies on general biology and infectious diseases. The main organoid models we have focused on are similar and include lung and brain organoids. The virus invasion mechanism is the most common focus in this field of research and has been the predominant focus of all previous reviews [23, 24, 26]. This review differs, however, for the following reasons: (1) we have summarised novel organoid models that have not been mentioned in previous reviews, such as the skin, salivary, and nasal organoid models; (2) we have summarised the limitations of the organoid models and the problems encountered in previous organoid cultures and have provided advice to help address these issues in future investigations; (3) we have described the applications of organoids in tracing the origins, mutations, and species specificity of SARS-CoV-2, thus providing a novel angle for SARS-CoV-2 studies; and (4) we have also discussed the different organoid culture methods and compared them to determine the most suitable culture environment. In conclusion, organoids are a reliable model for future medical research. In this review, we briefly defined the characteristics and advantages of organoids. We focused on their applications in SARS-CoV-2 infection, including mechanistic studies and drug and vaccine development. We summarised the research on tracing the SARS-CoV-2 origin and the characteristics of mutants, all of which were performed using organoid models. We also pointed out the limitations that currently exist and tried to provide a potential solution. In future research, we hope that stem cell-derived organoid models will be applied for the regeneration of injured epithelial cells in patients with COVID-19. Precision medicine is another potential demand for the treatment of patients with COVID-19; personalized organoids can be established to satisfy the individualised process of the disease. We believe that organoids will significantly contribute not only to SARS-CoV-2 studies but also to studies of other diseases. We expect that organoids will more closely resemble the in vivo environment and reflect human physiology with greater accuracy in future studies.
